# T126. PSYCHOTIC EXPERIENCES AND COMMON MENTAL DISORDERS IN CHILDHOOD AND ADOLESCENCE: BIDIRECTIONAL AND TRANSDIAGNOSTIC ASSOCIATIONS IN A LONGITUDINAL COMMUNITY-BASED STUDY

**DOI:** 10.1093/schbul/sby016.402

**Published:** 2018-04-01

**Authors:** Pedro Pan, Giovanni Salum, Felipe Argolo, Ary Gadelha, Rodrigo Bressan

**Affiliations:** 1 Universidade Federal de São Paulo; 2 Universidade Federal do Rio Grande do Sul

## Abstract

**Background:**

The prevalence of Psychotic Experiences (PE) in the general population is approximately 7%. Several studies report on the association of PE with non-psychotic mental disorders and dimensional psychopathology. However, few have addressed this relationship during adolescence using longitudinal data. Here, we aim to explore bidirectional associations of PE and common mental disorder in youth in a 3-year follow-up community-based study. We hypothesized that there is a link between PE and depression, corroborating findings from adult studies, and that mental disorders comorbidity significantly correlates to PE, showing a nonspecific effect of PE as a risk for a broad “psychiatric load/liability”.

**Methods:**

We analyzed data from the Brazilian High Risk Cohort (HRC), a large multi-site school-based study. At baseline, we evaluated 2,244 subjects (6–12 years old) using the Community Assessment of Psychotic Experiences (CAPE) and an adapted version of the Comprehensive Assessment of At-risk Mental States (CAARMS) by self-report and clinician ratings, respectively. Mental disorders in youth were assessed by the Development and Well-Being Assessment (DAWBA). We grouped mental disorders into 4 DSM-based categories: any depressive disorder, any anxiety disorder, any Attention Deficit Hyperactivity Disorder (ADHD), and any Oppositional Defiant Disorder or Conduct Disorder (ODD/CD). Subjects were reassessed after 3 years, with a retention rate of 75%. We used regression analyses to explore predictors of PE and mental disorders at follow-up. Finally, we investigated the bidirectional effect of PE as a nonspecific psychiatric “load/liability” by creating count variables for the number of comorbid psychiatric disorders for each participant. Poisson regression models tested the effect of PE (as a predictor) in the count variable (the outcome) controlling for potential confounders.

**Results:**

We found bidirectional associations between PE and mental disorders in youth. Baseline PE increased the risk of any depressive disorder at follow-up, and baseline ADHD was associated with PE at 3-year follow-up. Comorbidity analyses showed significant relationships in both directions, with an increased risk of PE according to the number of comorbid psychiatric disorders.

**Discussion:**

We showed that subthreshold psychotic symptoms predict subsequent depressive disorder, and nonspecifically relate to psychiatric comorbidity. These findings are concordant with the notion that psychotic experiences are part of the same psychiatric vulnerability conferred to common mental disorders, such as depression and ADHD.

Our results may inform future research on testing subclinical psychotic symptoms to further our understating on identifying high-risk groups for early intervention.

